# The role of *Acinetobacter baumannii* response regulator BfmR in pellicle formation and competitiveness via contact-dependent inhibition system

**DOI:** 10.1186/s12866-019-1621-5

**Published:** 2019-11-05

**Authors:** Renatas Krasauskas, Jūratė Skerniškytė, Julija Armalytė, Edita Sužiedėlienė

**Affiliations:** Institute of Biosciences, Life Sciences Center, Vilnius University, Vilnius, Lithuania

**Keywords:** *Acinetobacter*, BfmR, Pellicle, T6SS, CDI

## Abstract

**Background:**

*Acinetobacter baumannii* is one of the most important opportunistic pathogens responsible for hospital acquired infections. It displays multi-drug resistance profile and has the ability to colonize surfaces and persist under harsh conditions. *A. baumannii* two-component signal transduction system BfmRS, consisting of response regulator BfmR and sensor kinase BfmS, has been implicated in the control of various virulence-related traits and has been suggested to act as a global modulator of *A. baumannii* physiology.

**Results:**

Here, we assessed the role of BfmR regulator in pellicle formation and bacterial competition, features important for the establishment of *A. baumannii* in clinical environment. We show that BfmR is required for the pellicle formation of *A. baumannii*, as *ΔbfmRS* mutant lacked this phenotype. The loss of *bfmRS* also greatly reduced the secretion of *A. baumannii* Hcp protein, which is a component of T6SS secretion system. However, T6SS-mediated killing phenotype was not impaired in *ΔbfmRS* mutant. On the contrary, the same mutation resulted in the transcriptional activation of contact-dependent inhibition (CDI) system, which *A. baumannii* used to inhibit the growth of another clinical *A. baumannii* strain and a closely related species *Acinetobacter baylyi*.

**Conclusions:**

The obtained results indicate that BfmR is not only required for the pellicle phenotype induction in *A. baumannii*, but also, due to the down-regulation of a CDI system, could allow the incorporation of other *A. baumannii* strains or related species, possibly increasing the likelihood of the pathogens’ survival.

## Background

*Acinetobacter baumannii* is clinically important Gram-negative opportunistic pathogen responsible for the broad range of severe nosocomial infections in critically ill patients [1]. Due to its multi-drug resistance, the ability to form biofilms and various mechanisms allowing persistence under harsh environmental settings such as the presence of disinfectants, prolonged periods of desiccation, or oxidative stress, *A. baumannii* has become a threat to human health [2].

*A. baumannii* BfmR regulator along with histidine kinase BfmS, comprises two-component signal transduction system (TCS) BfmRS [3]. TCSs are widely distributed among prokaryotes allowing them to effectively adapt to ever-changing environment conditions. The membrane anchored sensor kinase, via phosphate transfer, controls its cognate response regulator. The latter, either regulates gene transcription directly or binds to target proteins eliciting a specific response of its host [4].

It has been shown that one of the clinically important *A. baumannii* traits, biofilm formation, is controlled by the BfmR [3]. Further studies provided some insights into the role of BfmRS in *A. baumannii* pathogenesis by showing that the loss of BfmS results in a significant reduction of motility [5]. The sensor BfmS has been shown to be required for the biofilm modulation, adhesion to epithelial cells, and increased sensitivity to serum killing [6]. The *bfmS* mutant showed increased secretion of membrane proteins, including OmpA, which is considered as one of the virulence factors of *A. baumannii* [6]. Further studies showed that BfmR is required for *A. baumannii* persistence in a murine lung infection model [7], for growth in human ascites fluid and for serum resistance [8, 9], while BfmS rather than BfmR was required for the successful growth in *Galleria mellonella* larvae [10].

BfmR has been also shown to control tolerance to desiccation and responses to oxidative stress [11]. In addition, via the up-regulation of β-lactamase production and cell envelope synthesis, resistance to β-lactams is also modulated by the BfmR [9]. Moreover, the whole *bfmRS* operon has been shown to be required for the regulation of the K locus, which is responsible for the capsular exopolysaccharide expression [12]. All observations discussed above indicate that BfmR is involved in the control of the genes responsible for a variety of phenotypes in *A. baumannii*, although its precise role is far from being fully elucidated.

In this study, we describe novel phenotypes that are reciprocally regulated by the BfmR. We demonstrate that the regulator is required for the pellicle formation, where bacteria form tightly packed biomass on the surface of culture media. At the same time, BfmR represses the contact-dependent inhibition (CDI) system, leading to the inability to suppress the growth of competing related strains. These observations suggest that *A. baumannii* via BfmR may modulate cooperative behavior against closely related strains during the pellicle formation, which may be advantageous for the establishment of this opportunistic pathogen in clinical environment.

## Results

### BfmR regulates *A. baumannii* pellicle formation

The pellicle is characterized as a form of biofilm that is floating on the surface of culture media and allows bacteria to acquire favorable ecological niche and directly access high concentrations of oxygen and nutrients from the air and liquid, respectively [13]. *A. baumannii* BfmR is responsible for the up-regulation of the *csuA/BABCDE* operon leading to the biofilm formation on solid surfaces [3]. It has also been determined that the CsuA/B pilin is the most abundant component of *A. baumannii* pellicle [14]. Therefore, we were interested whether the BfmR is involved in the manifestation of this phenotype.

From the collection of clinical *A. baumannii* isolates, characterized previously [15], the isolate V15, which showed a pellicle forming phenotype, has been selected. The deletion of *ΔbfmRS* operon was generated as described in the Methods. The decision to obtain a mutant with the deletion of the whole *bfmRS* operon was based on the previously published results indicating that the sensor kinase BfmS acts as a negative regulator of BfmR, and that only *bfmR* can fully complement whole *ΔbfmRS* mutant to WT levels [9]. The deletion was confirmed by sequencing and by performing qPCR analysis of *bfmR* and *bfmS* genes using total RNA. For complementation experiments, the plasmids carrying the genes encoding BfmR (p*bfmR*), BfmS (p*bfmS*), or both proteins (p*bfmRS*) were constructed and introduced into *A. baumannii* V15 as described in the Methods.

**Fig. 1:**
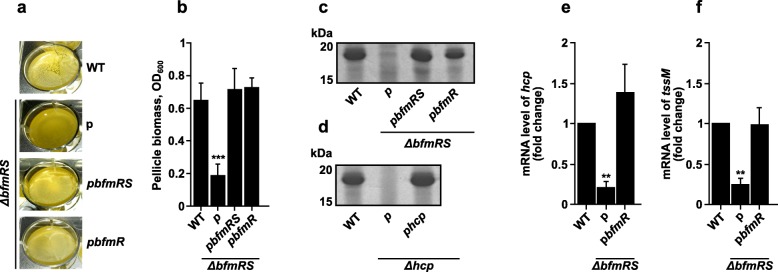
BfmR is required for *A. baumannii* pellicle formation and secretion of Hcp into culture media. **a** and **b** Pellicles formed by *A. baumannii* V15 (WT), *ΔbfmRS* mutant, and *ΔbfmRS* strain complemented with the plasmids p*bfmRS* or p*bfmR*: Macroscopic images of formed pellicles (**a**); Quantitative evaluation of pellicles with values normalized by the total growth volume (**b**). **c** and **d** 12.5% SDS-PAGE gel view of TCA-precipitated total protein fraction from culture media of WT, *ΔbfmRS*, and *ΔbfmRS* complemented with the plasmids p*bfmRS* or p*bfmR* (**c**); and WT, *Δhcp* mutant, and *Δhcp* complemented with a plasmid p*hcp* (**d**). Proteins were precipitated with TCA, separated by 12.5% SDS-PAGE, and visualized by staining with Coomassie blue. Numbers on the left denote molecular mass in kDa. **e** and **f** Expression levels of *A. baumannii* V15 transcripts *hcp* (**e**) and *tssM* (**f**) in WT, *ΔbfmRS,* and *ΔbfmRS* mutant complemented with p*bfmR*. Expression data in the graphs are displayed as a fold change compared to the WT which is set at 1. *hcp* gene in plasmid p*hc*p was induced using IPTG concentration of 0.1 mM. Error bars represent standard deviation. **, *p* < 0.01; ***, *p* < 0.001, relative to WT.

We then performed the pellicle formation assay by growing the strains in TSB media under the stationary conditions at 30 °C for 30 h as described in the Methods. These growth conditions were previously suggested to generally promote pellicle formation [13, 16]. Compared to the WT strain, which formed a thick and uniform pellicle morphology (Fig. 1a), the ability of *ΔbfmRS* mutant to develop a pellicle was impaired as only some biomass (white structures) was located near the walls of the wells (Fig. 1a and b). The inability of the mutant to form a pellicle was fully complemented with the *bfmR* allele or the whole *bfmRS* operon, when supplemented in trans (Fig. 1a and b). It must be noted that due to the toxicity of *bfmRS* and *bfmR* constructs with the native upstream sequences, we used plasmids with a leaky P*tac* promoter (transcripts were observed without additional supplementation of IPTG), which allowed the basal expression of *bfmR* at the level comparable to that of WT (approximately 2.86 ± 0.89 fold up-regulation compared to WT) and did not interfere with the growth of the strains. Finally, we observed no effect of the wild-type *bfmS* allele on the restoration of the pellicle phenotype in *ΔbfmRS* mutant, even using induction with IPTG up to 0.1 mM (Additional file 3: Figure S1a). These results show that BfmR is responsible for the pellicle phenotype manifestation, also they are consistent with the previously published data indicating that *ΔbfmRS* mutants may be complemented solely by the *bfmR* construct [9].

### Hcp secretion into the culture media is regulated by the BfmR

Pellicle formation requires various secreted proteins, polysacharides, and/or DNA to stabilize the structure [13]. Currently, only a few *A. baumannii* proteins have been linked to a pellicle formation [14]. However, there was an observation that during this process, the expression of multiple virulence factors changes [17]. To investigate, whether BfmR is involved in the regulation of pellicle phenotype-associated components, we first compared the electrophoretic profiles of precipitated total protein fractions from culture media of the WT and *ΔbfmRS* strains.

As can be seen in Fig. 1c, SDS-PAGE analysis of proteins precipitated from the culture media, showed a single band, migrating as a ~ 18 kDa entity, which was significantly reduced in the *ΔbfmRS* mutant. The secretion of the protein was restored after complementation with either *bfmRS* or *bfmR* alleles (Fig. 1c), correlating with the restoration of the pellicle phenotype. The band was identified by mass spectrometry as *A. baumannii* Hcp protein. The protein is a structural component of the bacterial Type VI secretion system (T6SS), which together with the additional proteins assembles into a needle-like apparatus that is used to puncture adjacent cells and to deliver effectors (toxins) into target cell [18, 19].

To confirm that the secreted protein was indeed Hcp, we generated *Δhcp* deletion in *A. baumannii* V15 strain, which resulted in the loss of Hcp secretion into culture media (Fig. 1d). The secretion was readily complemented with a copy of *hcp* gene cloned under the inducible promoter in the plasmid p*hcp*, when induced with 0.1 mM IPTG (Fig. 1d). The complementation under inducing conditions only, could be explained by the fact that before being secreted, the Hcp must assemble into tubular structure made from multiple copies of Hcp monomers [19].

The examination of the *hcp*-specific mRNA levels in the *ΔbfmRS* mutant showed an approximately five-fold reduction, when compared to the WT strain (Fig. 1e). The introduction of the plasmid p*bfmR* resulted in the restoration of transcription level. In parallel to the examination of *hcp* gene transcription, we assessed the transcription of *tssM* gene. The latter codes for the subunit of the membrane-anchoring complex, which is also essential for the assembly of T6SS apparatus [20]. As can be seen from the results presented in Fig. 1f, *tssM* mRNA levels in the *ΔbfmRS* were decreased four-fold, when compared to the WT. This indicates that the loss of *bfmR* might lead to the down-regulation of the whole T6SS system, resulting in the reduced secretion of Hcp into culture media.

The abundance of Hcp in culture media and the correlation between the Hcp secretion and the formation of pellicle exhibited by the WT strain, prompted us to test whether Hcp is required for the pellicle formation. The Hcp, secreted into media could be embedded into pellicle matrix, potentially reinforcing the structure. However, our results showed the deletion of *hcp* did not interfere with the manifestation of the phenotype (Additional file 3: Figure S1b). This shows that Hcp is not required for the pellicle formation of *A. baumannii*.

### Loss of *bfmRS* does not affect T6SS-mediated inter-genus killing

**Fig. 2:**
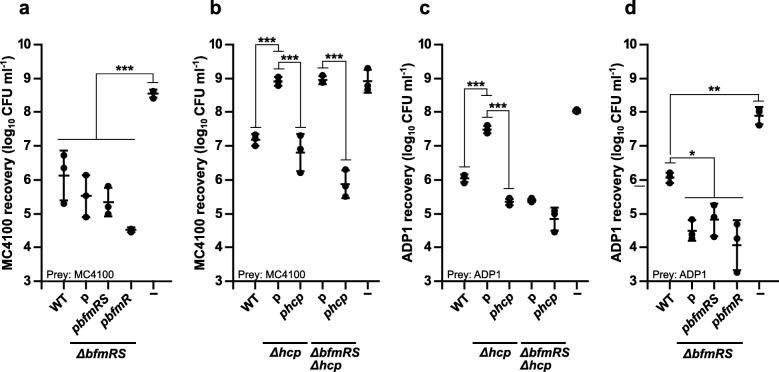
BfmR modulates *A. baumannii* V15 competitiveness against *E. coli* MC4100 and *A. baylyi* ADP1. Quantitative evaluation of inter-bacterial competition assay displaying a recovered number of prey: *E. coli* MC4100 (**a** and **b**) and *A. baylyi* ADP1 (**c** and **d**). Competition was performed with the following strains used as the aggressors: *A. baumannii* V15 (WT), *ΔbfmRS*, and *ΔbfmRS* complemented with the plasmids p*bfmRS* or p*bfmR* (**a** and **d**); WT, *Δhcp*, *Δhcp* strain complemented with the plasmid p*hcp*, *ΔbfmRSΔhcp* mutant, and *ΔbfmRSΔhcp* mutant complemented with the plasmid p*hcp* (**b** and **c**). *E. coli* DH5α was used as a negative non-competitive control to enumerate bacteria numbers if there were no competition. Error bars represent standard deviation. The horizontal lines represent mean value. Values were calculated from at least three independent experiments. *, *p* < 0.05; **, *p* < 0.01; ***, *p* < 0.001; n.s., not significant. *Hcp* gene in plasmid p*hcp* was induced using IPTG concentration of 0.1 mM

The findings above suggest that the down-regulation of T6SS might impact the killing phenotype of *A. baumannii* as it is known that Hcp secretion is the indication of a functional T6SS [18]. Previously, it has been demonstrated that *A. baumannii* is able to eliminate competing bacteria in a T6SS-dependent manner [21–24]. Therefore, we performed competition assays using *E. coli* MC4100 strain as a prey. Remarkably, while *A. baumannii* V15 was able to significantly reduce *E. coli* MC4100 numbers by 50–250-fold, the *ΔbfmRS* strain did not display any impairment in the killing phenotype (Fig. 2a). The phenotype remained mainly unchanged in the *ΔbfmRS* strain complemented with either *bfmRS*, or *bfmR* alleles (Fig. 2a). To confirm that the observed killing phenotype against *E. coli* was due to the function of T6SS, we investigated killing capacity of V15 *Δhcp* and the double mutant *ΔbfmRSΔhcp.* The results showed that both mutants exhibited reduction in killing of *E. coli* MC4100 (Fig. 2b and Additional file 4: Figure S2a). The phenotype of *Δhcp* and *ΔbfmRSΔhcp* mutants was readily complemented with the wild-type *hcp* allele (p*hcp*) under inducing (0.1 mM IPTG) conditions (Fig. 2b). We also evaluated whether there is a difference in killing efficiency against clinical strains of *Pseudomonas aeruginosa* (P16) and *Klebsiella pneumoniae* (K39). As can be seen in Additional file 5: Figure S3a-d, both strains can be killed via T6SS of *A. baumannii* V15. However, the results also show that the loss of *bfmRS*, apparently, does not influence the reduction of the killing efficiency of *A. baumannii*. These data indicate that despite clearly affecting the expression of the T6SS apparatus and significantly impairing the secretion of Hcp into the supernatant, the BfmRS system does not affect *A. baumannii* T6SS-mediated killing of *E. coli*, *P. aeruginosa* or *K. pneumoniae*.

### BfmR regulates T6SS-independent killing mechanism against closely related species

Next, we evaluated *A. baumannii* aggressiveness against more closely related species. For this purpose, we used *Acinetobacter baylyi* ADP1 strain as a prey. As can be seen in Fig. 2c, ADP1 strain was significantly (approximately 70–200-fold) out-competed by *A. baumannii* V15 and the killing was T6SS-dependent as the *Δhcp* mutant displayed significantly reduced killing efficiency (Fig. 2c). The killing phenotype of the mutant was readily restored to the WT level with the *hcp* allele under inducing conditions (Fig. 2c). Remarkably, in contrast to the results obtained with *E. coli* MC4100 strain, the double mutant *ΔbfmRSΔhcp*, lacking an active T6SS, still significantly reduced ADP1 numbers at the efficiency comparable to that of WT strain (Fig. 2c). Interestingly, we observed that the *ΔbfmRS* mutant was able to significantly reduce ADP1 numbers as well, and displayed even more aggressive killing phenotype than the WT (approximately 10-fold) (Fig. 2d). The observed killing phenotype of *ΔbfmRS* and *ΔbfmRSΔhcp* mutants could not be complemented with either p*bfmR* or p*bfmRS* (Fig. 2d and Additional file 4: Figure S2b). These results suggest, that *bfmRS* deletion leads to the activation of T6SS-independent killing mechanism that is effective against *A. baylyi* ADP1 but not *E. coli* MC4100.

### BfmR negatively regulates contact-dependent inhibition system of *A. baumannii*

We hypothesized that the observed killing of *A. baylyi* ADP1 but not *E. coli* MC4100 could be explained by the activation of currently poorly defined *A. baumannii* contact-dependent inhibition mechanism, which requires its receptor on the target cell and is classified as a type of Type V Secretion System (T5SS) and was shown to be functional in *A. baumannii* [25, 26].

CDI systems are composed of three components belonging to the *cdiBAI* gene cluster. The first two genes (*cdiB* and *cdiA*) encode a two-partner secretion system, which allows a large CdiA hemagglutinin-repeat protein to be displayed on the surface of bacterial cell. The last gene (*cdiI*) encodes an immunity protein, which binds and neutralizes the cognate toxin [25]. Previous work indicated that *Acinetobacter sp*. might contain up to two functional CDI systems [26, 27].

**Fig. 3:**
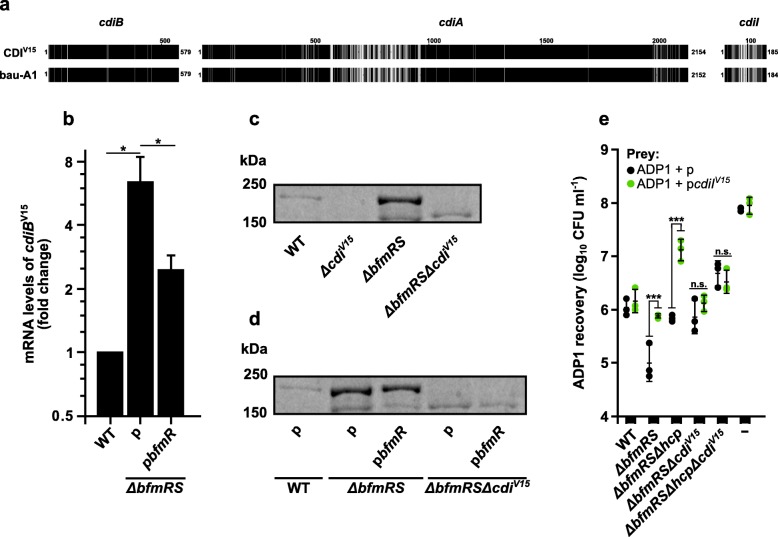
*A. baumannii* V15 contains a functional CDI system. **a** Multiple amino acid sequence alignment of each of the CDI^V15^ loci encoded proteins with the most similar type-I CDI system (bau-A1/Genbank accession number: AMFH01000034.1). The most similar CDI was determined by performing multiple sequence comparisons with the recently characterized *Acinetobacter* CDI systems [28]. Alignments are displayed as sequence fingerprints using alignment shading software Texshade (version 1.25) [29]. Identical amino acids are shaded black. Unique residues are shaded gray. Annotations above alignments indicate the aligned CDI proteins. **b** Expression of *A. baumannii cdiB* in WT, *ΔbfmRS*, and *ΔbfmRS* mutant complemented with p*bfmR*. The data are displayed as a fold change compared to the WT which is set at 1. **c** and **d** Part of 10% SDS-PAGE gel view showing the presence of the CdiA protein found in the total protein fraction from culture media of WT, *Δcdi*^*V15*^, *ΔbfmRS*, *ΔbfmRSΔcdi*^*V15*^, and *ΔbfmRS* and *ΔbfmRSΔcdi*^*V15*^ complemented with p*bfmR.* Proteins were precipitated with TCA, separated by 10% SDS-PAGE, and visualized by staining with Coomassie blue. Numbers on the left denote molecular mass in kDa. **e** Quantitative evaluation of inter-bacterial competition assay displaying a recovered number of *A. baylyi* ADP1 with or without plasmid p*cdiI*^*V15*^ containing *A. baumannii* V15 immunity gene *cdiI*. Competition was performed with *A. baumannii* V15 mutants *ΔbfmRS*, *ΔbfmRSΔhcp*, *ΔbfmRSΔcdi*^*V15*^, *ΔbfmRSΔhcpΔcdi*^*V15*^ as the aggressors. *E. coli* DH5α was used as a negative non-competitive control to enumerate bacteria numbers if there were no competition. Error bars represent standard deviation. The horizontal lines represent mean value. Values were calculated from at least three independent experiments. *, *p* < 0.05; ***, *p* < 0.001; n.s., not significant. *cdiI*^*V15*^ gene in plasmid p*cdiI*^*V15*^ was induced using IPTG concentration of 5 mM

Therefore, based on the previous classification of *A. baumannii* CDI systems [28], we created a set of 3 primer pairs targeting a rather conserved *cdiB* genes and managed to identify and sequence the CDI locus of *A. baumannii* V15 strain (CDI^V15^). The results indicated that CDI^V15^ is a type-I CDI system with a CdiA protein, most identical to bau-A1/pit-A3 type CdiA proteins (90%) (Fig. 3a). Other CDI^V15^ proteins, namely CdiB and CdiI, were 98% and 66% identical to their counterparts in bau-A1 system, respectively (Fig. 3a). It is worth to note that some *A. baumannii* strains, namely AR_0037 (GenBank accession: MPBX01000005.1/bau-D9 CdiA type [28]), 1295549 (JFXB01000002.1/bau-B2), 426863 (JFYF01000002.1/bau-B2), ATCC19606 (JMRY01000015.1/bau-B2) contained nearly identical immunity proteins in the genome regions, which did not have a CDI system nearby, indicating that these strains are potentially immune to CDI^V15^.

Having identified that *A. baumannii* V15 contains an intact *cdi* locus, we further investigated, whether the observed T6SS-independent killing mechanism is indeed caused by the CDI^V15^ system. By comparing the mRNA levels of the *cdiB* gene between *A. baumannii* V15 *ΔbfmRS* mutant and WT we observed that the mutant displayed increased transcript levels of *cdiB* by ~ 6.4-fold. Additionally, complementation of *ΔbfmRS* mutant with the *bfmR* allele displayed a ~ 2.5-fold increase in the transcript levels of *cdiB*, when compared to the WT (Fig. 3b). This indicates that the complemented strain displayed an intermediate transcription level of the CDI^V15^. This result was consistent with the observed killing phenotype displayed against *A. baylyi* ADP1 strain, where the aggressiveness level of *ΔbfmRS* mutant could not be complemented with the *bfmR* allele to that of WT.

### *A. baumannii* uses CDI to out-compete *A. baylyi* ADP1

To confirm our observation that the CDI^V15^ system is activated in the *bfmRS* mutant, a partial deletion of *A. baumannii cdi*^*V15*^ locus was generated in the WT and *ΔbfmRS* strains. In the resulting mutants, the *cdiB* gene was left intact but approximately 90% of the *cdiA*^*V15*^ along with *cdiI*^*V15*^ were deleted. This was due to the size of the genomic region that prevented us from obtaining the whole CDI^V15^ operon deletion. It is interesting to note, that during the characterization of *A. baumannii ΔbfmRSΔcdiV15* and *Δcdi*^*V15*^ mutants, we have observed the loss of ~ 200 kDa band in the SDS-PAGE gel of precipitated total protein fraction from culture media (Fig. 3c). The band corresponds to the predicted molecular weight of CdiA protein (~ 231 kDa), and its identity was subsequently confirmed by mass spectrometry. We have also noticed that, consistently with the mRNA data (Fig. 3b), the predicted CdiA band in WT was of lower intensity when compared to the *ΔbfmRS* strain (Fig. 3c). Additionally, *ΔbfmRS* strain complemented with the p*bfmR* displayed the predicted CdiA band of intermediate intensity, compared to the WT and *ΔbfmRS* (Fig. 3d).

We then investigated the aggressiveness of the mutants against *A. baylyi* ADP1. In addition, a plasmid containing the *cdiI*^*V15*^ allele under the inducible promoter was created and introduced into ADP1 strain. The *cdiI*^*V15*^ codes for the putative immunity gene, which should protect the host from the aggressor, if the latter uses the CDI^V15^ system for the killing. By performing the killing assays, we found that the deletion of *cdi*^*V15*^ in the *ΔbfmRS* and *ΔbfmRSΔhcp* mutants resulted in a low (~ 10-fold) but significant reduction of aggressiveness, when compared to the parent mutant (Fig. 3e). Interestingly, *ΔbfmRSΔhcpΔcdi*^*V15*^ mutant still displayed a killing phenotype (Fig. 3e). The reasons that caused this are currently unknown. However, it could be explained by the activation of a secondary CDI system that we were unable to detect with our PCR screen, as it is known that some *Acinetobacter* sp. strains contain two active CDI systems [26, 27].

The introduction of the *cdiI*^*V15*^ allele into *A. baylyi* ADP1 strain reduced the susceptibility to the killing by *A. baumannii ΔbfmRSΔhcp* and *ΔbfmRS*, but not by the *ΔbfmRSΔcdi*^*V15*^ and *ΔbfmRSΔhcpΔcdi*^*V15*^ strains (Fig. 3e), further confirming our findings that the *ΔbfmRS* and *ΔbfmRSΔhcp* mutants activate T6SS-independent killing mechanism, and show that this phenotype could be attributed to the activation of CDI^V15^ locus.

### CDI-mediated *A. baumannii* intra-species competition

Bacterial inter-species killing via CDI system was observed only in a few cases and showed a very low efficiency, therefore it was suggested that the primary role of CDI is to differentiate sibling cells from other closely related bacteria from the same species [30, 31]. This could explain a rather modest changes in the killing efficiency that we observed towards *A. baylyi* ADP1. This prompted us to investigate the CDI-mediated killing phenomenon within the *A. baumannii* species. For this purpose, using primer pairs targeting *cdiB* genes of all known *A. baumannii* CDI systems, we have screened clinical *A. baumannii* isolates, representing different genotypically related groups (pulsotypes) of strains (*n* = 15) belonging to international clonal lineage II (IC II) by PCR [32]. The results showed that two clinical *A. baumannii* strains II-g and II-h did not contain a known *cdiB* gene. Therefore, these strains were selected for further competition experiments.

**Fig. 4:**
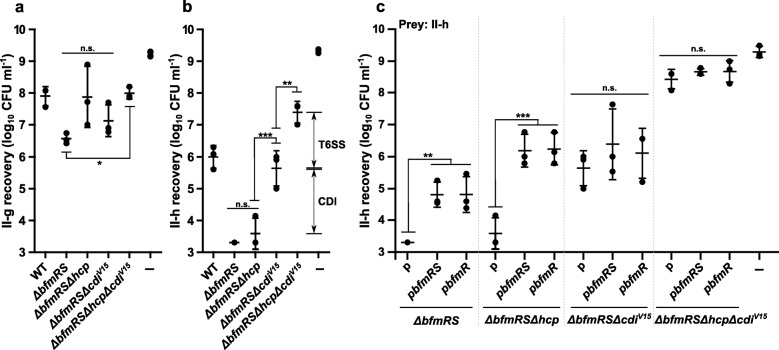
CDI mediated *A. baumannii* intra-species competition. **a**-**c** Quantitative evaluation of inter-bacterial competition assay displaying a recovered number of *A. baumannii* clinical strains II-g (**a**) and II-h (**b** and **c**). Competition was performed with the following *A. baumannii* V15 mutants used as the aggressors: *ΔbfmRS*, *ΔbfmRSΔhcp*, *ΔbfmRSΔcdi*^*V15*^, *ΔbfmRSΔhcpΔcdi*^*V15*^ (**a** and **b**). The II-h strain was also challenged with the *ΔbfmRS*, *ΔbfmRSΔhcp*, *ΔbfmRSΔcdi*^*V15*^, *ΔbfmRSΔhcpΔcdi*^*V15*^ mutants, which were complemented with either p*bfmRS* or p*bfmR* (**c**). *E. coli* DH5α was used as a negative non-competitive control to enumerate bacteria numbers if there were no competition. Error bars represent standard deviation. The horizontal lines represent mean value. Values were calculated from at least three independent experiments. ***, *p* < 0.001; **, *p* < 0.01; n.s., not significant

As can be seen in Fig. 4a and b, II-h strain was highly susceptible to the killing by *A. baumannii* V15, while II-g strain displayed a low susceptibility to this phenotype (Fig. 4a and Additional file 6: Figure S4a). The total numbers of the susceptible strain II-h were reduced ~ 10^7^-fold by the *ΔbfmRS* and *ΔbfmRSΔhcp* mutants (Fig. 4b). When the competition was performed with the triple mutant *ΔbfmRSΔhcpΔcdi*^*V15*^, the recovery of II-h increased by a factor of 10^4^, compared to *ΔbfmRS*. The strain containing only functional T6SS (*ΔbfmRSΔcdi*^*V15*^), displayed an intermediate phenotype (Fig. 4b). It is worth to mention that the WT displayed mostly T6SS-dependent killing phenotype (Additional file 6: Figure S4b). These results indicate, that the *ΔbfmRS* mutant kills II-h strain via both mechanisms – T6SS and CDI, while WT strain uses only T6SS-dependent killing.

Additionally, we investigated, whether the complementation with the *bfmR* allele resulted in an inhibition of the CDI-mediated killing against II-h. Notably, the results showed that when *ΔbfmRS* mutant was complemented with either p*bfmR* or p*bfmRS*, the recovery numbers of II-h increased by ~ 30-fold (Fig. 4c)*.* Interestingly, when the *ΔbfmRSΔhcp* mutant was complemented with the same alleles, the reduction of killing phenotype was ~ 500-fold (Fig. 4c). Lastly, as expected, the complementation of either *ΔbfmRSΔcdi*^*V15*^ or *ΔbfmRSΔhcpΔcdi*^*V15*^ with the *bfmR* or *bfmRS* alleles did not influence any change in the killing phenotype of the mutants (Fig. 4c). These results were consistent with the observed intermediate transcriptional up-regulation of CDI^V15^ system in the *ΔbfmRS* strain complemented with the *bfmR*, when compared to the WT and *ΔbfmRS* strains (Fig. 3b)*.* Additionally, *bfmR* or *bfmRS* complemented strains *ΔbfmRSΔhcp*, *ΔbfmRSΔcdi*^*V15*^, containing either functional CDI or T6SS, respectively, displayed an intermediate killing phenotype, compared to the *ΔbfmRS* and *ΔbfmRSΔhcpΔcdi*^*V15*^ (Fig. 4c). These results confirm observation that BfmR acts as a negative regulator of the CDI^V15^ system of *A. baumannii* V15.

## Discussion

In this study, we aimed to further characterize the response regulator BfmR and its role in *A. baumannii* physiology. We were able to show that the presence of BfmR induces pellicle formation, while at the same time the regulator acts negatively on the contact-dependent inhibition system. The ability to form pellicle varies among *A. baumannii* strains considerably [16, 32] and seems to be affected by the production of secondary signaling molecule cAMP [16], indicating that regulatory mechanisms play a great role in the manifestation of this phenotype. Given the fact that pellicle formation is considered as an important factor for the persistence and transmission of the pathogenic species [13, 14], the understanding of how it is regulated in *A. baumannii* is important. Our results, showing that BfmR is responsible for the formation of this structure, suggests that there is a specific currently unknown signal or signals that the BfmRS system responds to, which leads to the induction of the pellicle. Currently, the best known inducer of the pellicle phenotype is oxygen gradient that emerges when bacteria grow to a high cell density [13]. Also, the fact that BfmR is responsible for the surface biofilm formation [3] and the observation that pellicles were slightly attached to the walls of the tubes [14] suggests that surface biofilms may be the initial stage of pellicle formation. This speculation is also based on the previous findings showing that the four-day mature pellicle, compared to the one-day pellicle, contains ~ 4.5-fold increased BfmR levels [17], indicating that during pellicle formation the levels of the protein gradually increase. Since we were unable to reliably dissect pellicle and biofilm formation phenotypes apart, we could not reject the possibility that pellicle formation is a secondary effect due to the inability to form surface biofilms.

Biofilm formation is characterized by some level of bacterial organization, that includes morphologically and genetically diverse individuals, which generate and respond to signaling by the nearby cells and/or the surroundings [33, 34]. Since biofilm is a densely packed structure, competition for limited resources and space occurs. In bacteria, the antagonism is exerted mainly via two different contact-dependent inter-bacterial competition systems: non-specific type VI secretion system (T6SS) and receptor-specific contact-dependent growth inhibition system (CDI) [35].

Our results show that *A. baumannii* BfmR is involved in the regulation of both of them. Firstly, we determined that BfmR is required for the extensive secretion of Hcp protein into media and up-regulates transcription of *hcp* and *tssM* genes, which are essential for T6SS activity [20]. However, despite the fact that Hcp secretion is the hallmark of a functional Type VI secretion system [18], we did not observe the reduction in T6SS-dependent killing phenotype. This suggests that either the inhibition of killing effect was too small, or the Hcp secretion plays a role in other, killing independent activities such as metal ion acquisition or in contribution to pathogenesis [36–38]. Our results are consistent with the previous observations indicating that a mature four-day *A. baumannii* pellicle displays up-regulation of some T6SS locus genes, compared to the planktonic cultures [17]. This suggests that during the unfavourable conditions *A. baumannii* may choose to activate T6SS. The presence of this phenotype in the pellicle could be crucial for bacteria to defend from other competing species and prevent from the rise of mutants in a population, which do not contribute to the secretion of stabilizing materials. However, there seems to be a great heterogeneity among different *A. baumannii* strains in terms of ability to secrete Hcp and in T6SS regulation mechanisms in general [20, 22, 23, 39].

We also determined that the BfmR negatively regulates yet poorly characterized contact-dependent inhibition system. Such systems, allow some species of Gram-negative bacteria to deliver toxic effectors to neighboring bacteria and inhibit their growth [25, 26, 40]. It is known that CDI requires a receptor to deliver a toxin to its target, therefore the inhibitory activity is restricted to closely related bacteria, and, according to some suggestions, could be used to distinguish self from non-self [35, 41]. Our results are consistent with these observations as we detected CDI activity against *A. baumannii* and *A. baylyi* ADP1 but not against *E. coli* MC4100, *P. aeruginosa*, and *K. pneumoniae*.

The negative regulation of the CDI^V15^ system by the BfmR suggests that CDI is not required for the pellicle/biofilm formation of *A. baumannii* V15. Indeed, our data show that the deletion of CDI^V15^ did not impact the ability of the strain to form a pellicle or biofilm (unpublished observation). Our results support previous findings indicating that *A. baylyi* ADP1 also does not require CDI system to form biofilms [27]. Recently, evolution experiments with *P. aeruginosa* indicated that biofilm cultures, compared to the planktonic bacteria, experienced higher mutability rates, which led to the diversification of biofilm population with clone variants that contributed to the population’s ability to colonize the surface [34]. The turned off CDI system in these populations could allow their preservation while at the same time the active T6SS could prevent the invading species.

It must be noted that out of two clinical *A. baumannii* isolates, which were selected for the competition experiments, only one was susceptible to the CDI^V15^-mediated killing, despite both of them lacking CDI cluster and belonging to the same international clonal lineage II [42, 43]. The resistant strain may possess similar orphan immunity gene, which could protect it from the CDI^V15^ system, as we observed the presence of these orphan genes dispersed throughout at least some of the *A. baumannii* strains. Additionally, we noticed, that these regions may contain more than one immunity module (unpublished observation), indicating that some strains may posses innate immunity protecting them from the attack executed by a strain containing a different CDI system. Moreover, our recent work indicates that the susceptible strain II-h is capsule-deficient [32]. This observation could also explain the susceptibility phenotype as the required receptor for the CDI-mediated killing may be hidden in the capsule-positive strain II-g, thereby effectively restricting the *A. baumanii* V15 CDI^V15^ attack.

## Conclusions

It is well known that BfmRS system promotes biofilm formation which is an important feature of microbial persistence under unfavourable conditions. However, the regulatory pathways of *A. baumannii* global regulator BfmR are still poorly understood. Here we provide evidence that *A. baumannii* via the BfmR, is able to promote pellicle formation, and Hcp secretion into culture media. However, at the same time, BfmR acts as a negative regulator of a CDI system of *A. baumannii*, which is used to inhibit the growth of related bacteria. Therefore, our results suggest that during these conditions *A. baumannii* via the BfmRS system may allow a cooperative behavior towards related bacteria, which could improve the survival chances of *A. baumannii*.

## Methods

### Bacterial strains and growth conditions

*E. coli* JM107 was used for all cloning experiments and DNA manipulations. *Bacillus* spp. was used as the source for *sacB* gene. *Acinetobacter* sp., *Pseudomonas aeruginosa* (P16), and *Klebsiella pneumoniae* (K39) strains were grown in tryptic soy broth (TSB) (Oxoid). All *E. coli* strains were grown in Luria-Bertani (LB) media. All *A. baumannii* strains used in the work were previously characterized [15, 32]. Bacteria were grown at 37 °C, unless indicated otherwise. Growth media was supplemented with antibiotics, where appropriate: ampicillin 100 μg mL^− 1^, gentamicin 10 μg mL^− 1^, streptomycin 100 μg mL^− 1^, ceftazidime 10 μg mL^− 1^. Bacterial strains and plasmids used in this study are listed in the Additional file 1: Table S1.

### Plasmid construction

All molecular biology procedures were performed using reagents obtained from Thermo Fisher Scientific and according the manufacturer’s recommendations. Primers were obtained from Metabion or Thermo Fisher Scientific and are listed in the Additional file 2: Table S2. All final constructs were verified by sequencing. The suicide plasmid pUC19_*sacB* was constructed by amplifying the levansucrase gene *sacB* using primers sacB_F/sacB_R and cloning the PCR product into pUC19 via XbaI and PaeI. The *A. baumannii*/*E. coli* IPTG inducible shuttle expression plasmid pUC_gm_AcORI_*Ptac*_*gfp*_TER was obtained by cloning of *Ptac* promoter, terminator sites (TER) (both from pKK223–3), and the *gfp* gene (from pAcGFP1-C3) into the pUC19 plasmid containing *Acinetobacter* sp. ORI from pWH1266 [44]. The plasmid was then inverse amplified with primers M13_rwd/Aac3I_seqR to remove the gentamicin cassette and blunt ligated to the *lacI*^*q*^ gene amplified from BL21(DE3) strain genomic DNA to generate *A. baumannii*/*E. coli* shuttle expression plasmid pUC_AcORI_*Ptac*_*gfp*_TER_*lacI*^*q*^2.

Complementation plasmids were constructed by replacing the *gfp* gene in the IPTG inducible shuttle expression plasmid with the required gene amplified from *A. baumannii* V15 genomic DNA. Control plasmid pUC_AcORI_*Ptac*_TER_*lacI*^*q*^2 was obtained by removing the *gfp* gene and is denoted in figures as “p”, where relevant. The complementation plasmid containing the *cdiI*^*V15*^ gene was further modified by replacing the *bla* gene with the *aac3I* cassette. The created plasmids were used to complement the relevant strains of *A. baumannii* (Additional file 1: Table S1).

### Generation of *A. baumannii* mutant strains

A modified marker-less gene deletion technique from Oh et al. [45] was used to obtain mutant strains of *A. baumannii*. Approximately 1 kb long upstream and downstream regions of the genes to be deleted were amplified separately from the genomic DNA of *A. baumannii* V15 and joined via the overlap PCR with gentamicin resistance cassette *aac3I* using primer pairs described in the Additional file 2: Table S2. The resulting DNA fragments were cloned into pUC19_*sacB* plasmid. The relevant *A. baumannii* strains were electroporated with the obtained plasmids and selected on LB agar plates with 10 μg mL^− 1^ of gentamicin. Then, a single colony was inoculated into LB media without antibiotics and grown overnight at 37 °C with shaking. Serial dilutions of the overnight culture were plated onto LB agar plates containing 10% sucrose and grown overnight at 37 °C. Mutants were identified by PCR with specific primers (Additional file 2: Table S2) and confirmed by sequencing. All obtained mutants and their variants complemented with various constructs were tested for growth impairments by inoculating overnight cultures of *A. baumannii* into the wells of 96-well polystyrene plates at a density of 10^6^ CFU mL^− 1^ in 0.25x TSB and then measuring growth at 37 °C until stationary phase. IPTG were added at the early logarithmic phase (OD_600_ = 0.25–0.3) where necessary to determine the maximum concentration that did not induce growth impairments due to the presence of constructs.

### Pellicle formation assays

The pellicle formation was evaluated by inoculating overnight cultures of *A. baumannii* grown in 1x TSB media at 37 °C into the wells of a flat-bottom 12 well polystyrene microplate at a density of 10^6^ CFU mL^− 1^ in a total volume of 3 mL. The cultures were incubated stationary at 30 °C for 30 h. To collect the pellicles from the surface of the media, 200 μL of isopropanol were added to the each well, which allowed to easily remove almost all pellicle material. The removed pellicles were resuspended in 500 μL of 10 mM NaOH, followed by a quick neutralization with HCl and the OD_600_ of suspension was measured and normalized to the total volume of culture so as to be comparable to the planktonic OD_600_ readings.

### Protein secretion assay

Total protein content from culture media used in the pellicle formation assay was precipitated using 100% (w/v) trichloroacetic acid (TCA) as follows. Firstly, the collected culture media was centrifuged at 13000 g for 10 min at 4 °C and subsequently filtered through 0.22 μm filter to remove remaining biomass. Then, in the resulting supernatant TCA was added to a final concentration of 10% (w/v), and centrifuged at 13000 g for 45 min at 4 °C. The resulting pellet was washed twice with ice-cold acetone and dried by incubating tube at 95 °C for a few minutes before being re-suspended with Laemmli-SDS-PAGE sample buffer. The samples were analyzed using the Laemmli-SDS-PAGE system. After electrophoresis, gels were stained with Coomassie brilliant blue. Approximately 3.5 μg of the total protein were loaded into each well. The PageRuler™ unstained broad range protein ladder (7.5 μl) was used as marker. The whole gels are shown in Additional file 7: Figure S5a-S5d. Protein identification by MALDI-TOF mass spectrometry was undertaken at Proteomics Department of Vilnius University Life Sciences Center.

### RNA isolation and analysis of gene expression by qPCR

Overnight cultures of *A. baumannii* grown in 1x TSB media at 37 °C were diluted with 0.25x TSB and inoculated into the wells of a flat-bottom 96 well polystyrene microplate at a density of 10^6^ CFU mL^− 1^ in 0.25x TSB. The cultures were grown until the logarithmic phase (OD_600_ = 0.35–0.4). Total RNA was isolated, DNA removed and cDNA synthesized as recommended by the supplier (Thermo Fisher Scientific). RNA integrity and contamination with DNA was checked by agarose gel electrophoresis. qPCR was performed using primer pairs listed in the Additional file 2: Table S2 (all primers exhibited 95–107% amplification efficiency (with > 0.99 coefficient of determination) at used concentrations). Product specificity was investigated by melting curve analysis. The changes in gene expression were calculated as ΔΔC_t_, using *rpoB* as a house-keeping gene. At least three biological replicates, each with two technical replicates, were performed.

### Inter-bacterial competition assay

The assay was performed as described previously [26] with some modifications. Briefly, the strains grown overnight in TSB media at 37 °C were washed twice with the fresh TSB to remove residual antibiotics. Then, bacteria were diluted with the fresh TSB to a final concentration of ~ 10^8^ CFU mL^− 1^ and mixed at aggressor (*A. baumannii* V15):prey ratio of 10:1, 10:1, 20:1, when the competition was performed with *E. coli* strain MC4100, *A. baylyi* strain ADP1, or all other strains, respectively. Five microlitre of resulting suspension was placed onto TSB media containing 1.5% agar and allowed to dry. The competitions were performed at 37 °C for 6 h. To quantitatively evaluate the number of surviving bacteria, spots were excised from the plate, vigorously re-suspended in TSB broth, serially diluted, and plated on TSB agar plates containing selective antibiotics: streptomycin (100 μg mL^− 1^) for *E. coli* strain MC4100, ampicillin (100 μg mL^− 1^) for all *A. baumannii* V15 mutants, ceftazidime (10 μg mL^− 1^) for all other clinical *A. baumannii* strains, gentamicin (10 μg mL^− 1^) for *A. baylyi* ADP1, *P. aeruginosa*, and *K. pneumoniae*. All strains had a natural resistance or were transformed with a plasmid containing appropriate marker allowing for a selective isolation. All experiments contained control reactions, which consisted of non-aggressive *E. coli* strain DH5α mixed with each of the strain to obtain a total number bacteria if there were no competition between strains. The obtained number of colonies was calculated as CFU per mL of culture.

### Statistical analyses

All statistical comparisons were performed using one-way ANOVA (*p* = 0.05) with a Tukey HSD post-hoc test. Inter-bacterial competition assay was calculated as follows: CFU per mL was normalized by taking first the decadic logarithm and using these values for statistical analysis. Changes in gene expression experiments were considered significant if the differences were more than 2-fold. Asterisks in the figures denote the statistically significant difference between the groups (n.s., not significant; *, *p* < 0.05; **, *p* < 0.01; ***, *p* < 0.001). The analyses were performed using R package (version 3.2.3). Graphs were drawn using QtiPlot.

### GenBank accession number

The sequence of *A. baumannii* V15 *cdiBAI* locus has been deposited in GenBank under the accession number MK405474.

## Supplementary information

**Additional file 1: Table S1.** Bacterial strains and plasmids used in the study.

**Additional file 2: Table S2.** Primers used in the study.

**Additional file 3: Figure S1.** BfmS and functional T6SS are not required for pellicle production. Quantitative evaluation of pellicles formed by: (a) *A. baumannii* V15 (WT), *ΔbfmRS* mutant, and *ΔbfmRS* mutant, complemented with the plasmid p*bfmS*; (b) WT, *Δhcp* mutant, and *Δhcp* mutant, complemented with the plasmid p*hcp*. Pellicle values were normalized by the total growth volume. Error bars represent standard deviation. IPTG denotes induction conditions using 0 or 0.1 mM IPTG concentration.

**Additional file 4: Figure S2.** Loss of BfmRS system activates T6SS-independent killing phenotype of *A. baumannii* V15 against *A. baylyi* ADP1 only. Quantitative evaluation of inter-bacterial competition assay displaying a recovered number of prey: (a) *E. coli* MC4100 and (b) *A. baylyi* ADP1. Competition was performed with the following strains used as the aggressors: WT, *ΔbfmRSΔhcp* mutant, *ΔbfmRSΔhcp* strain complemented with the plasmids p*bfmRS* or p*bfmR*. *E. coli* DH5α was used as a negative non-competitive control to enumerate bacteria numbers if there were no competition. Error bars represent standard deviation. The horizontal lines represent mean value. Values were calculated from at least three independent experiments. ***, *p* < 0.001; n.s., not significant.

**Additional file 5: Figure S3.** Quantitative evaluation of inter-bacterial competition assays displaying the recovered numbers of clinical strains that were used as a prey: (a and b) *Pseudomonas aeruginosa* P16 and (c and d) *Klebsiella pneumoniae* K39. Competition was performed with the following strains used as the aggressors: WT, *ΔbfmRS, Δhcp*, *ΔbfmRSΔhcp.* The *hcp* mutants were also complemented with the wild-type *hcp* allele (plasmid p*hcp*). *E. coli* DH5α was used as a negative non-competitive control to enumerate bacteria numbers if there were no competition. Error bars represent standard deviation. The horizontal lines represent mean value. Values were calculated from at least three independent experiments.*, *p* < 0.05; **, *p* < 0.01; ***, *p* < 0.001; n.s., not significant. The *hcp* gene in plasmid p*hcp* was induced using IPTG concentration of 0.1 mM.

**Additional file 6: Figure S4.** T6SS is used as the main mechanism for species antagonism by *A. baumannii* V15 (WT) strain. Quantitative evaluation of inter-bacterial competition assay displaying a recovered number of CDI lacking *A. baumannii* strains used as a prey: (a) II-g; (b) II-h. Competition was performed with the following strains used as the aggressors: WT, *Δhcp* mutant, *Δhcp* mutant complemented with the wild-type *hcp* allele (plasmid p*hcp*). *E. coli* DH5α was used as a negative non-competitive control to enumerate bacteria numbers if there were no competition. Error bars represent standard deviation. The horizontal lines represent mean value. Values were calculated from at least three independent experiments.*, *p* < 0.05; n.s., not significant. *Hcp* gene in plasmid p*hcp* was induced using IPTG concentration of 0.1 mM.

**Additional file 7: Figure S5.** The whole gels showing TCA-precipitated total protein fraction from culture media. Related to Fig. 1c-d and Fig. 3c-d. Proteins separated by 12.5% (a and b) or 10% (c and d) SDS-PAGE and visualized by staining with Coomassie blue. PageRuler™ Unstained Broad Range Protein Ladder (Thermo Fisher) was used as marker. Numbers on the left of the gels denote molecular mass in kDa.

## Data Availability

The sequence of *A. baumannii* V15 *cdiBAI* locus has been deposited in GenBank under the accession number MK405474.

## Abbreviations

cAMP: Cyclic adenosine monophosphate
CDI: Contact-dependent inhibition system
cDNA: Complementary DNA
CFU: Colony Formation Unit
HCl: Hydrochloric acid
IC: International clonal lineage
IPTG: Isopropyl β-D-thiogalactopyranoside
kb: Kilobase
kDa: Kilodalton
LB: Luria-Bertani media
MALDI-TOF: Matrix-assisted laser desorption/ionization time of flight mass spectrometry
NaOH: Sodium hydroxide
PCR: Polymerase Chain Reaction
qPCR: Quantitative polymerase chain reaction
SDS-PAGE: Sodium dodecyl sulphate-polyacrylamide gel electrophoresis
T5SS: Type V Secretion System
T6SS: Type VI Secretion System
TCA: Trichloroacetic acid
TCS: Two-component signal transduction system
TSB: Tryptic soy broth
w/v: Weight by volume
WT: Wild-type

## References

[1] Antunes LCS, Visca P, Towner KJ (2014). Acinetobacter baumannii: evolution of a global pathogen. Pathog Dis.

[2] Harding CM, Hennon SW, Feldman MF (2018). Uncovering the mechanisms of *Acinetobacter baumannii* virulence. Nat Rev Microbiol.

[3] Tomaras AP, Flagler MJ, Dorsey CW, Gaddy JA, Actis LA (2008). Characterization of a two-component regulatory system from *Acinetobacter baumannii* that controls biofilm formation and cellular morphology. Microbiology..

[4] Zschiedrich Christopher P., Keidel Victoria, Szurmant Hendrik (2016). Molecular Mechanisms of Two-Component Signal Transduction. Journal of Molecular Biology.

[5] Clemmer KM, Bonomo RA, Rather PN (2011). Genetic analysis of surface motility in *Acinetobacter baumannii*. Microbiology..

[6] Liou M-L, Soo P-C, Ling S-R, Kuo H-Y, Tang CY, Chang K-C (2014). The sensor kinase BfmS mediates virulence in *Acinetobacter baumannii*. J Microbiol Immunol Infect.

[7] Wang N, Ozer EA, Mandel MJ, Hauser AR. Genome-wide identification of *Acinetobacter baumannii* genes necessary for persistence in the lung. MBio. 2014;5(3):e01163–14. 10.1128/mBio.01163-14 PMC4049102 24895306

[8] Russo TA, Manohar A, Beanan JM, Olson R, MacDonald U, Graham J, et al. The response regulator BfmR is a potential drug target for *Acinetobacter baumannii*. mSphere. 2016;1(3):e00082-16. 10.1128/mSphere.00082-16 PMC4888885 27303741

[9] Geisinger E, Mortman NJ, Vargas-Cuebas G, Tai AK, Isberg RR (2018). A global regulatory system links virulence and antibiotic resistance to envelope homeostasis in *Acinetobacter baumannii*. PLoS Pathog.

[10] Gebhardt MJ, Gallagher LA, Jacobson RK, Usacheva EA, Peterson LR, Zurawski DV (2015). Joint transcriptional control of virulence and resistance to antibiotic and environmental stress in *Acinetobacter baumannii*. MBio..

[11] Farrow JM, Wells G, Pesci EC (2018). Desiccation tolerance in *Acinetobacter baumannii* is mediated by the two-component response regulator BfmR. PLoS One.

[12] Geisinger E, Isberg RR (2015). Antibiotic modulation of capsular exopolysaccharide and virulence in *Acinetobacter baumannii*. PLoS Pathog.

[13] Armitano J, Méjean V, Jourlin-Castelli C (2014). Gram-negative bacteria can also form pellicles. Environ Microbiol Rep.

[14] Nait Chabane Y, Marti S, Rihouey C, Alexandre S, Hardouin J, Lesouhaitier O (2014). Characterisation of pellicles formed by *Acinetobacter baumannii* at the air-liquid interface. PLoS One.

[15] Povilonis J, Seputiene V, Krasauskas R, Juskaite R, Miskinyte M, Suziedelis K (2013). Spread of carbapenem-resistant *Acinetobacter baumannii* carrying a plasmid with two genes encoding OXA-72 carbapenemase in Lithuanian hospitals. J Antimicrob Chemother.

[16] Giles SK, Stroeher UH, Eijkelkamp BA, Brown MH (2015). Identification of genes essential for pellicle formation in *Acinetobacter baumannii*. BMC Microbiol.

[17] Kentache T, Ben Abdelkrim A, Jouenne T, Dé E, Hardouin J (2017). Global dynamic proteome study of a pellicle-forming *Acinetobacter baumannii* strain. Mol Cell Proteomics.

[18] Pukatzki S, McAuley SB, Miyata ST (2009). The type VI secretion system: translocation of effectors and effector-domains. Curr Opin Microbiol.

[19] Brunet YR, Hénin J, Celia H, Cascales E (2014). Type VI secretion and bacteriophage tail tubes share a common assembly pathway. EMBO Rep.

[20] Weber BS, Miyata ST, Iwashkiw JA, Mortensen BL, Skaar EP, Pukatzki S (2013). Genomic and functional analysis of the type VI secretion system in *Acinetobacter*. PLoS One.

[21] Carruthers MD, Nicholson PA, Tracy EN, Munson RS (2013). Acinetobacter baumannii utilizes a type VI secretion system for bacterial competition. PLoS One.

[22] Repizo GD, Gagné S, Foucault-Grunenwald M-L, Borges V, Charpentier X, Limansky AS (2015). Differential role of the T6SS in *Acinetobacter baumannii* virulence. PLoS One.

[23] Weber BS, Ly PM, Irwin JN, Pukatzki S, Feldman MF (2015). A multidrug resistance plasmid contains the molecular switch for type VI secretion in *Acinetobacter baumannii*. Proc Natl Acad Sci U S A.

[24] Weber BS, Hennon SW, Wright MS, Scott NE, de Berardinis V, Foster LJ, et al. Genetic dissection of the type VI secretion system in *Acinetobacter* and identification of a novel peptidoglycan hydrolase, TagX, required for its biogenesis. MBio. 2016;7(5):e01253-16. 10.1128/mBio.01253-16 PMC5061870 27729508

[25] Willett JLE, Ruhe ZC, Goulding CW, Low DA, Hayes CS (2015). Contact-dependent growth inhibition (CDI) and CdiB/CdiA two-partner secretion proteins. J Mol Biol.

[26] Harding CM, Pulido MR, Venanzio GD, Kinsella RL, Webb AI, Scott NE (2017). Pathogenic *Acinetobacter* species have a functional type I secretion system and contact-dependent inhibition systems. J Biol Chem.

[27] De Gregorio E, Esposito EP, Zarrilli R, Di Nocera PP (2018). Contact-dependent growth inhibition proteins in *Acinetobacter baylyi* ADP1. Curr Microbiol.

[28] De Gregorio E, Zarrilli R, Di Nocera PP (2019). Contact-dependent growth inhibition systems in *Acinetobacter*. Sci Rep.

[29] Beitz E (2000). TeXshade: shading and labeling of multiple sequence alignments using LaTeX2e. Bioinformatics..

[30] Beck CM, Morse RP, Cunningham DA, Iniguez A, Low DA, Goulding CW (2014). CdiA from *Enterobacter cloacae* delivers a toxic ribosomal RNase into target bacteria. Structure..

[31] Koskiniemi S, Garza-Sánchez F, Edman N, Chaudhuri S, Poole SJ, Manoil C (2015). Genetic analysis of the CDI pathway from *Burkholderia pseudomallei* 1026b. PLoS One.

[32] Skerniškytė J, Krasauskas R, Péchoux C, Kulakauskas S, Armalytė J, Sužiedėlienė E (2019). Surface-related features and virulence among *Acinetobacter baumannii* clinical isolates belonging to international clones I and II. Front Microbiol.

[33] Poltak SR, Cooper VS (2011). Ecological succession in long-term experimentally evolved biofilms produces synergistic communities. ISME J.

[34] Flynn KM, Dowell G, Johnson TM, Koestler BJ, Waters CM, Cooper VS (2016). Evolution of ecological diversity in biofilms of *Pseudomonas aeruginosa* by altered cyclic Diguanylate signaling. J Bacteriol.

[35] Garcia EC (2018). Contact-dependent interbacterial toxins deliver a message. Curr Opin Microbiol.

[36] Kapitein N, Mogk A (2013). Deadly syringes: type VI secretion system activities in pathogenicity and interbacterial competition. Curr Opin Microbiol.

[37] Lin J, Zhang W, Cheng J, Yang X, Zhu K, Wang Y (2017). A *Pseudomonas* T6SS effector recruits PQS-containing outer membrane vesicles for iron acquisition. Nat Commun.

[38] Wang J, Zhou Z, He F, Ruan Z, Jiang Y, Hua X (2018). The role of the type VI secretion system vgrG gene in the virulence and antimicrobial resistance of *Acinetobacter baumannii* ATCC 19606. PLoS One.

[39] Di Venanzio G, Moon KH, Weber BS, Lopez J, Ly PM, Potter RF (2019). Multidrug-resistant plasmids repress chromosomally encoded T6SS to enable their dissemination. Proc Natl Acad Sci U S A.

[40] Ruhe ZC, Low DA, Hayes CS (2013). Bacterial contact-dependent growth inhibition. Trends Microbiol.

[41] Ruhe ZC, Wallace AB, Low DA, Hayes CS. Receptor polymorphism restricts contact-dependent growth inhibition to members of the same species. MBio. 2013;4(4):e00480-13. 10.1128/mBio.00480-13 PMC3735181 23882017

[42] Turton JF, Gabriel SN, Valderrey C, Kaufmann ME, Pitt TL (2007). Use of sequence-based typing and multiplex PCR to identify clonal lineages of outbreak strains of *Acinetobacter baumannii*. Clin Microbiol Infect.

[43] Karah N, Sundsfjord A, Towner K, Samuelsen Ø (2012). Insights into the global molecular epidemiology of carbapenem non-susceptible clones of *Acinetobacter baumannii*. Drug Resist Updat.

[44] Armalytė J, Jurėnas D, Krasauskas R, Čepauskas A, Sužiedėlienė E (2018). The higBA toxin-antitoxin module from the opportunistic pathogen *Acinetobacter baumannii* – regulation, activity, and evolution. Front Microbiol.

[45] Oh MH, Lee JC, Kim J, Choi CH, Han K (2015). Simple method for Markerless gene deletion in multidrug-resistant *Acinetobacter baumannii*. Appl Environ Microbiol.

