# Supplementary material for: The role of Acinetobacter baumannii response regulator BfmR in pellicle formation and competitiveness via contact-dependent inhibition system
Source: BMC Microbiol. 2019 Nov 5;19:241. doi: 10.1186/s12866-019-1621-5 (PMC6833216; doi:10.1186/s12866-019-1621-5)
Supplement: Supplementary file 1 — Additional file 1: Table S1. Bacterial strains and plasmids used in the study. [file 12866_2019_1621_MOESM1_ESM.pdf]

Table S1. Bacterial strains and plasmids used in the study.

Strain or plasmid	Description and/or genotype	Reference
Escherichia coli		
JM107	General laboratory strain used for all cloning experiments and DNA manipulations. *endA1*, *glnV44*, *thi*$^-$, *relA1*, *gyrA96*, *Δ(lac-proAB)* [F', *traD36*, *proAB*$^+$, *lacI*^{q}*ZΔM15*], *hsdR17*(R_K^- m_K^+), *λ*$^-$.	[1]
DH5α	Strain used for inter-bacterial competitive growth assays as a control. *F-, Δ(argF-lac)169, φ80dlacZ58(M15), ΔphoA8, glnX44(AS), λ*$^-$*, deoR481, rfbC1, gyrA96(NalR), recA1, endA1, thiE1, hsdR17*.	[2]
MC4100	Strain used for inter-bacterial competitive growth assays as prey F-, [*araD139*]$_{B/r}$, *Δ(argF-lac)169*, *λ-*, *e14-*, *flhD5301*, *Δ(fruK-yeiR)725(fruA25)*, *relA1*, *rpsL150(strR)*, *rbsR22*, *Δ(fimB-fimE)632(::IS1)*, *deoC1*.	[3]
BL21(DE3)	Genomic DNA used as a template for the amplification of lacI^q F-, *lon-11*, *Δ(ompT-nfrA)885*, *Δ(galM-ybhJ)884*, *λDE3 [lacI, lacUV5-T7 gene 1, ind1, sam7, nin5]*, *Δ46*, *[mal*$^+$*]*$_{K-12}$(λ^S), *hsdS10*.	[4]
Bacillus subtilis	Used for the amplification of the *sacB* gene.	Gift from Audrius Gegeckas
Acinetobacter sp.		
V15	Clinical isolate, IC_other, unique pulsotype U18.	[5]
V15 *ΔbfmRS*	V15 derivative with deleted *bfmRS* operon.	This study
V15 *ΔbfmRS pbfmRS*	V15 *ΔbfmRS* complemented with pUC_AcORI_*Ptac_bfmRS*_TER_*lacI*^{q}2.	This study
V15 *ΔbfmRS pbfmR*	V15 *ΔbfmRS* complemented with pUC_AcORI_*Ptac_bfmR*_TER_*lacI*^{q}2.	This study
V15 *ΔbfmRS pbfmS*	V15 *ΔbfmRS* complemented with pUC_AcORI_*Ptac_bfmS*_TER_*lacI*^{q}2.	This study
V15 *Δhcp*	V15 derivative with deleted *hcp* gene.	This study
V15 *Δhcp phcp*	V15 *Δhcp* complemented with pUC_AcORI_*Ptac_hcp*_TER_*lacI*^{q}2.	This study
V15 *ΔbfmRSΔhcp*	V15 *ΔbfmRS* derivative with deleted *hcp* gene.	This study

V15 *ΔbfmRSΔhcp pbfmRS*	V15 *ΔbfmRSΔhcp* complemented with pUC_AcORI_*Ptac_bfmRS*_TER_*lacI^{q}2*.	This study
V15 *ΔbfmRSΔhcp pbfmR*	V15 *ΔbfmRSΔhcp* complemented with pUC_AcORI_*Ptac_bfmR*_TER_*lacI^{q}2*.	This study
V15 *Δcdi^{V15}*	V15 derivative with partial deletion of *cdiBAI* operon.	This study
V15 *ΔhcpΔcdi^{V15}*	V15 *Δhcp* derivative with partial deletion of *cdiBAI* operon.	This study
V15 *ΔbfmRSΔcdi^{V15}*	V15 *ΔbfmRS* derivative with partial deletion of *cdiBAI* operon.	This study
V15 ΔbfmRSΔhcpΔcdi^{V15}	V15 *ΔbfmRSΔhcp* derivative with partial deletion of *cdiBAI* operon.	This study
V15 *ΔbfmRSΔcdi^{V15} pbfmRS*	V15 *ΔbfmRSΔcdi^{V15}* complemented with pUC_AcORI_*Ptac_bfmRS*_TER_*lacI^{q}2*.	This study
V15 *ΔbfmRSΔcdi^{V15} pbfmR*	V15 *ΔbfmRSΔcdi^{V15}* complemented with pUC_AcORI_*Ptac_bfmR*_TER_*lacI^{q}2*.	This study
V15 *ΔbfmRSΔhcpΔcdi^{V15} pbfmRS*	V15 *ΔbfmRSΔhcpΔcdi^{V15}* complemented with pUC_AcORI_*Ptac_bfmRS*_TER_*lacI^{q}2*.	This study
V15 *ΔbfmRSΔhcpΔcdi^{V15} pbfmR*	V15 *ΔbfmRSΔhcpΔcdi^{V15}* complemented with pUC_AcORI_*Ptac_bfmR*_TER_*lacI^{q}2*.	This study
A. baylyi ADP1		ATCC 33305
A. baylyi ADP1 *pcdiI^{V15}*	*A. baylyi* ADP1 strain complemented with pUC_AcORI_*Ptac_cdiI^{V15}*_TER_*lacI^{q}2_gm*.	This study
ABV36/II-a1	Clinical *A. baumannii* isolate, IC II, pulsotype II-a1	[5,6]
AB52/II-a	Clinical *A. baumannii* isolate, IC II, pulsotype II-a	
ABV63/II-a2	Clinical *A. baumannii* isolate, IC II, pulsotype II-a2	
ABS11/II-a1	Clinical *A. baumannii* isolate, IC II, pulsotype II-a1	
ABS08/II-a3	Clinical *A. baumannii* isolate, IC II, pulsotype II-a3	
AB15/II-b	Clinical *A. baumannii* isolate, IC II, pulsotype II-b	
AB60/II-c	Clinical *A. baumannii* isolate, IC II, pulsotype II-c	
AB330/II-d	Clinical *A. baumannii* isolate, IC II, pulsotype II-d	
ABV22/II-d	Clinical *A. baumannii* isolate, IC II, pulsotype II-d	
AB323/II-e	Clinical *A. baumannii* isolate, IC II, pulsotype II-e	
AB141/II-f	Clinical *A. baumannii* isolate, IC II, pulsotype II-f	
AB106/II-g	Clinical *A. baumannii* isolate, IC II, pulsotype II-g	
AB109/II-h	Clinical *A. baumannii* isolate, IC II, pulsotype II-h	

AB288/II-j2	Clinical *A. baumannii* isolate, IC II, pulsotype II-j2	
AB282/II-j4	Clinical *A. baumannii* isolate, IC II, pulsotype II-j4	
**Other**		
Pseudomonas aeruginosa P16	Clinical isolate	[7]
Klebsiella pneumoniae K39	Clinical isolate	[8]
**Plasmids**		
pUC19	Used as a scaffold for the construction of sucide pUC19_*sacB*; Amp^{R}.	[9]
pWH1266	*Acinetobacter calcoaceticus* plasmid fragment containing *ori* cloned to pBR322	[10]
pUC19_*sacB*	*A. baumannii* suicide vector containing *sacB* gene from *Bacillus* spp. cloned via *Xba*I and *Pae*I; Amp^{R}.	This study
pUC19_sacB_*bfmRS*UPDwn_gmR	pUC19_*sacB* derivative with *ΔbfmRS*::*aac3I*; Amp^{R}; Gm^{R}.	This study
pUC19_sacB_*hcp*UPDwn_gmR	pUC19_*sacB* derivative with *Δhcp*::*aac3I*; Amp^{R}; Gm^{R}.	This study
pUC_gm	*A. baumannii aac3I* gene cloned into pUC19; Amp^{R}; Gm^{R}.	This study
pUC_gm_AcORI	*Acinetobacter sp. ori* cloned to pUC19_gm; Amp^{R}; Gm^{R}.	This study
pUC_gm_AcORI_*gfp*	pUC_gm_AcORI derivative with *gfp* gene from pAcGFP1-C3 cloned downstream to *Acinetobacter sp. ori*; Amp^{R}, Gm^{R}.	This study
pUC_gm_AcORI_*Ptac_gfp*	pUC_gm_AcORI_*gfp* derivative with *Ptac* from pKK223-3 cloned downstream to *Acinetobacter sp.* ori; Amp^{R}; Gm^{R}.	This study
pUC_gm_AcORI_*Ptac_gfp_TER*	pUC_gm_AcORI_*Ptac_gfp* derivative with terminator (TER) sequence from pKK223-3 cloned downstream *gfp* gene; Amp^{R}; Gm^{R}.	This study
pUC_AcORI_*Ptac_gfp*_TER_*lacI*^{q}*2*	pUC_gm_AcORI_*Ptac_gfp_TER* derivative with *lacI*^{q} downstream the terminator sequence, replacing *aac3I*; Amp^{R}.	This study
pUC_AcORI_*Ptac*_TER_*lacI*^{q}*2*	pUC_AcORI_*Ptac_gfp*_TER_*lacI*^{q}*2* derivative where *gfp* gene is removed; Amp^{R}.	This study
pUC_AcORI_*Ptac_bfmRS*_TER_*lacI*^{q}*2*	pUC_AcORI_*Ptac_gfp*_TER_*lacI*^{q}*2* derivative where *gfp* gene is replaced with *bfmRS* operon; Amp^{R}.	This study

pUC_AcORI_*Ptac_bfmR*_TER_*lacI^{q}2*	pUC_AcORI_*Ptac_gfp*_TER_*lacI^{q}2* derivative where *gfp* gene is replaced with wild-type *bfmR* allele; Amp^R.	This study
pUC_AcORI_*Ptac_bfmS*_TER_*lacI^{q}2*	pUC_AcORI_*Ptac_gfp*_TER_*lacI^{q}2* derivative where *gfp* gene is replaced with wild-type *bfmS* allele; Amp^R.	This study
pUC_AcORI_*Ptac_hcp*_TER_*lacI^{q}2*	pUC_AcORI_*Ptac_gfp*_TER_*lacI^{q}2* derivative where *gfp* gene is replaced with *hcp* gene; Amp^R.	This study
pUC_AcORI_*Ptac_imm^{V15}*_TER_*lacI^{q}2*	pUC_AcORI_*Ptac_gfp*_TER_*lacI^{q}2* derivative where *gfp* gene is replaced with *cdiI^{V15}* gene; Amp^R.	This study
pUC_AcORI_*Ptac_cdiI^{V15}*_TER_*lacI^{q}2_gm*	pUC_AcORI_*Ptac_imm^{V15}*_TER_*lacI^{q}2* derivative where *bla* is replaced with *aac3I;* Gm^R.	This study
pAcGFP1-C3	Used as a source for *gfp* gene; Kan^R.	Clonetech laboratories Inc.
pKK223-3	Used as a source for *Ptac* promoter and termination sites; Amp^R.	Cloning Vector from PL-Pharmacia. GenBank:M77749.1

Amp^R, ampicillin resistant; Gm^R, gentamicin resistant; Kan^R, kanamycin resistant

References

1. Yanisch-Perron C, Vieira J, Messing J. Improved M13 phage cloning vectors and host strains: nucleotide sequences of the M13mp18 and pUC19 vectors. Gene. 1985;33(1):103-19.
2. Woodcock DM, Crowther PJ, Doherty J, Jefferson S, DeCruz E, Noyer-Weidner M, et al. Quantitative evaluation of *Escherichia coli* host strains for tolerance to cytosine methylation in plasmid and phage recombinants. Nucleic Acids Res. 1989 11;17(9):3469-78.

3. Casadaban MJ, Cohen SN. Lactose genes fused to exogenous promoters in one step using a Mu-lac bacteriophage: in vivo probe for transcriptional control sequences. Proc Natl Acad Sci U S A. 1979;76(9):4530-3.
4. Wood WB. Host specificity of DNA produced by *Escherichia coli*: bacterial mutations affecting the restriction and modification of DNA. J Mol Biol. 1966;16(1):118-33.
5. Povilonis J, Šeputienė V, Krasauskas R, Juškaitė R, Miškinytė M, Sužiedėlis K, et al. Spread of carbapenem-resistant *Acinetobacter baumannii* carrying a plasmid with two genes encoding OXA-72 carbapenemase in Lithuanian hospitals. J Antimicrob Chemother. 2013;68(5):1000-6.
6. Skerniškytė J, Krasauskas R, Péchoux C, Kulakauskas S, Armalytė J, Sužiedėlienė E. Surface-Related Features and Virulence Among *Acinetobacter baumannii* Clinical Isolates Belonging to International Clones I and II. Front Microbiol. 2019;9:3116.
7. Krasauskas R, Labeikytė D, Markuckas A, Povilonis J, Armalytė J, Plančiūnienė R, Kavaliauskas P, Sužiedėlienė E. Purification and characterization of a new β-lactamase OXA-205 from *Pseudomonas aeruginosa*. Ann Clin Microbiol Antimicrob. 2015;14:52.
8. Seputiene V, Linkevicius M, Bogdaite A, Povilonis J, Planciūniene R, Giedraitiene A, Pavilonis A, Suziedeliene E. Molecular characterization of extended-spectrum β-lactamase-producing *Escherichia coli* and *Klebsiella pneumoniae* isolates from hospitals in Lithuania. J Med Microbiol. 2010;59(Pt 10):1263-5
9. Norrander J, Kempe T, Messing J. Construction of improved M13 vectors using oligodeoxynucleotide-directed mutagenesis. Gene. 1983;26(1):101-6.
10. Hunger M, Schmucker R, Kishan V, Hillen W. Analysis and nucleotide sequence of an origin of DNA replication in *Acinetobacter calcoaceticus* and its use for *Escherichia coli* shuttle plasmids. Gene. 1990;87(1):45-51.
