# Supplementary material for: The role of Acinetobacter baumannii response regulator BfmR in pellicle formation and competitiveness via contact-dependent inhibition system
Source: BMC Microbiol. 2019 Nov 5;19:241. doi: 10.1186/s12866-019-1621-5 (PMC6833216; doi:10.1186/s12866-019-1621-5)
Supplement: Supplementary file 2 — Additional file 2: Table S2. Primers used in the study. [file 12866_2019_1621_MOESM2_ESM.pdf]

Table S2. Primers used in the study.

Oligonucleotide	Oligonucleotide sequence (5'→3')	Description
P-Ab-ITSF/<br>P-Ab-ITSB	CATTATCACGGTAATTAGTG/<br>AGAGCACTGTGCACTTAAG	*A. baumannii* specific identification primers [1]
sacB_F/<br>sacB_R	GTTGTCTAGAGATCCTTTTTAACCCATCAC/<br>GTTGGCATGCTGGGATTCACCTTTATGTTG	Amplification of *sacB* gene from *Bacillus subtilis*. *Xba*I and *Pae*I(*Sph*I) restriction sites are underlined
BfmR_Ptac_F/<br>BfmR_Ptac_R	CATGAGCCAAGAAGAAAAGTTACC/<br>TTACAATCCATTGGTTTCTTTAAC	Amplification of *bfmRS* operon, wild-type *bfmR*, and mutant *bfmR*(D58A). Cloning into inducible vector
BfmS_Ptac_R	GAACCTGATGCAACTCAG	Amplification of *bfmS* or *bfmRS* operon. Cloning into inducible vector
BfmS_Ptac_F	CGTGTTTAAACACAGTATATTCCTGC	Amplification of *bfmS* and cloning into inducible vector
hcp_compl_F/<br>hcp_compl_R	CATGAAAGATATATACGTTGAGTTTCGC/<br>CTTTATGTCAGCCTCCACCAA	Amplification of *hcp*. Cloning into inducible vector
BfmR_F	GTTGAAGCTTAAATGCAGCAACATCTCC	Verification for the *ΔbfmRS* deletion. Sequence obtained from [2]
ter_F/<br>ter_R	GGGCCATGGGCATGCGGTACCCTGTTTTGGCGGATGAGAG/<br>GTTGGAGCTCTTTGTAGAAACGCAAAAAGGC	Amplification of transcription termination site from pKK223-3 vector. *Kpn*I and *Sac*I restriction

		sites are underlined.
M13_rwd/ Aac3I_seqR	ACTGGCCGTCGTTTTAC/ CGAAGTCGAGGCATTTCTGT	Inverted amplification of pUC_gm_AcORI_Ptac_AcGFP_ter to remove Gm^R
LacIq2_R/ LacIq2_F	CTCACTGCCCGCTTTCCA/ ATCGAATGGTGCAAAAC	Amplification of *$lacI^q$* gene.
BfmR01F/ BfmRS01R	TCACGCATTGCACCATAA/ GGAACCTGATGCAACTCAGTTATAAATCATTGCCCCTATAAATCTC	Amplification of *bfmRS* upstream region. Overlay with *bfmRS* downstream region is underlined
BfmS02F/ BfmS02Rgm	TTATAACTGAGTTGCATCAGG/ CGTTCAAGCCGAGATGAATTCGATCGGCCGAATTTGGTTATTG	Amplification of *bfmRS* downstream region. Overlay with *aac3I* downstream region is underlined
5_hcpFwd/ 5_hcpRev	TCAGGAAACGCCTTCAAATC/ CTTTATGTCAGCCTCCACCAAGCTGACCTTGATTAATTTGAGG	Amplification of *hcp* upstream region. Overlay with *hcp* downstream region is underlined
3_hcpFwd/ 3_hcpRev	TTGGTGGAGGCTGACATAAAG/ CGTTCAAGCCGAGATGAATTCGATCGCTCAAATTCGGATACATGCTG	Amplification of *hcp* downstream region. Overlay with *aac3I* downstream region is underlined
GentR_F/ GentR_R	GATCGAGCTCAGGACAGAAATGCCTCGACT/ GATCGAATTCATCTCGGCTTGAACGAATTG	Amplification of *aac3I* gene *Sac*I and *Eco*RI restriction sites

		are underlined
Bfm_check_F/<br>Bfm_check_R	CAACACCCTGAGATTTACCG/<br>CAGCAACTTTTGTGCCTATG	Verification for the Δ*bfmRS* deletion
hcp_checkF/<br>hcp_checkR	GTCAACTTGGCGTGGTCTTT/<br>TGGGGTTCAGCATATTTTTCA	Verification for the Δ*hcp* deletion
Hcp_seq_chk	TGCTTCTGCTGGAAATGTTG	Verification for the Δ*hcp* deletion
rpoB_qF/<br>rpoB_qR	CGATTCGTACAGAACATTCTT/<br>TAAAGCAGCATTGCCAGAATA	qPCR house-keeping gene primer
T6hcF/<br>T6hcR	ACTTCAAGTAGTGTGGGCGG/<br>AAGTCCACTCAACAGCAGCA	qPCR primer for quantification of *hcp* expression
TssMF/<br>TssMR	TGCTTTGGCGCAGTAAGACA/<br>CTTGCTGTGCGGATACAACG	qPCR primer for quantification of *tssM* expression
CDI_5Fwd_XH858/<br>CDI1R/<br>CDIF/<br>CDI_3Rev_XH858/<br>CDI_seq_2_F/<br>CDI_seq_2_R/<br>CDI_compl_F/<br>CDI_seq4_F/<br>CDI_seq4_R/<br>CDI_seq5_R/<br>CDI_seq_7F/<br>CDI_seq_7R/	TTGTCGGTACGACTGCTGCT/<br>TGCTGTCAGGTGCAATCAGC/<br>GGTAAATGGCCGCAATAGCATAC/<br>TGGCCGTGGAGCTTTTAACT/<br>GGGGCGAATAATGTCAGTGC/<br>CTCAACACGGCAAGCAGATA/<br>CATGTTAACTAAGAACTTTATAACTTCTTC/<br>AATATCAATCTTGGGGAAGGTC/<br>TCACTGGCATAGATTTGACTC/<br>CTTAAGCCAATATTCTGAGCA/<br>TTCAAGTGGTGGTTTAGTGCTC/<br>CAGAAGGATTAGGACCATCACC/	Sequencing of *cdiBAI*^{V15} locus of *A. baumannii* V15
CDI_5Fwd_final_short/<br>CDI_5Rev_final_short	GCTGATTGCACCTGACAGCA/<br>TAAACGACCTGTAATAGACCGCACTGACAT TATTCGCCCC	Amplification of *cdiBAI* upstream region. Overlay with *cdiBAI*^{V15} downstream region is underlined.
		Sequencing of *cdi* locus of *A. baumannii* V15

CDI_3Fwd_final/<br>CDI_3Rev_final_gm	GGTCTATTACAGGTCGTTTACTTTAAATAG/<br>CGTTCAAGCCGAGATGAATTCGATCACCCCAAATCTTACTCCAATCG	Amplification of *cdiBAI*^{V15} downstream region. Overlay with *aac3I* downstream region is underlined
Cdi_Imm_F/<br>Cdi_Imm_R	TTAAAGTAAACGACCTGTAATAGACC/<br>CATGATCGATTTTGTTAAAGAATTATCTGC	Amplification of *cdiI*^{V15}. Cloning into inducible vector.
CDI1F/<br>CDI1R	GGTAAATGGCCGCAATAGCATAC/<br>TGCTGTCAGGTGCAATCAGC	Detection of Type I CDI systems among *A. baumannii*; and for the quantification of *cdiBAI*^{V15} expression
CDI2F/<br>CDI2R	TTTATGCTTCGGGCAATCTGG/<br>GCACACCAAGTCGCAAAGAA	Detection of Type I CDI systems among *A. baumannii*
F_CDI_cluster3_4/<br>R_CDI_cluster3_4	CGGAGAGGTTGGTGGAAAAACTGC/<br>CAAAGGCGCACCCACAAAAGC	Detection of Type II CDI system among *A. baumannii*

Gm^{R}, gentamicin resistance.

References

1. Chen T-L, Sin L-K, Wu RC-C, Shaio M-F, Huang L-Y, Fung C-P, et al. Comparison of one-tube multiplex PCR, automated ribotyping and intergenic spacer (ITS) sequencing for rapid identification of *Acinetobacter baumannii*. Clin Microbiol Infect. 2007;13(8):801-6.

2. Tomaras AP, Flagler MJ, Dorsey CW, Gaddy JA, Actis LA. Characterization of a two-component regulatory system from *Acinetobacter baumannii* that controls biofilm formation and cellular morphology. Microbiology. 2008;154(Pt 11):3398-409.
