# Supplementary figures and images for: The role of Acinetobacter baumannii response regulator BfmR in pellicle formation and competitiveness via contact-dependent inhibition system
Source: BMC Microbiol. 2019 Nov 5;19:241. doi: 10.1186/s12866-019-1621-5 (PMC6833216; doi:10.1186/s12866-019-1621-5)

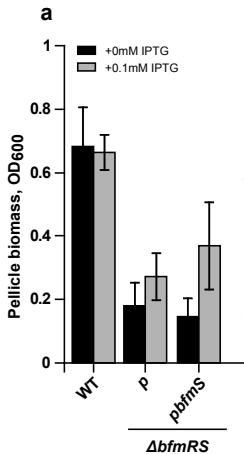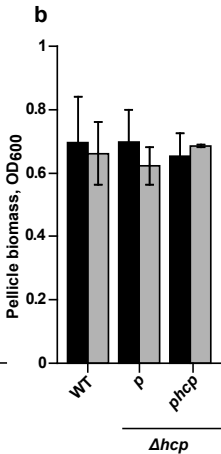

Supplement: Supplementary file 3 — Additional file 3: Figure S1. BfmS and functional T6SS are not required for pellicle production. Quantitative evaluation of pellicles formed by: (a) A. baumannii V15 (WT), ΔbfmRS mutant, and ΔbfmRS mutant, complemented with the plasmid pbfmS; (b) WT, Δhcp mutant, and Δhcp mutant, complemented with the plasmid phcp. Pellicle values were normalized by the total growth volume. Error bars represent standard deviation. IPTG denotes induction conditions using 0 or 0.1 mM IPTG concentration. [file 12866_2019_1621_MOESM3_ESM.pdf]

**a**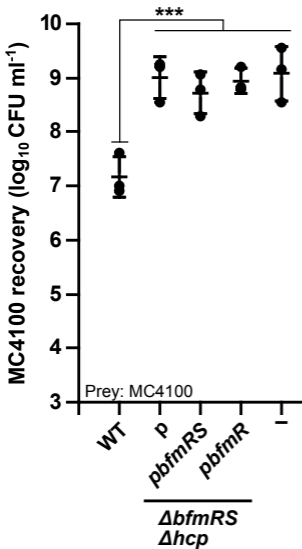**b**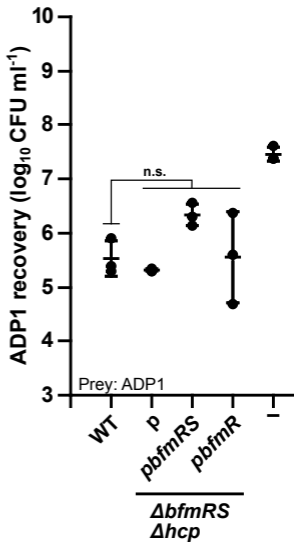

Supplement: Supplementary file 4 — Additional file 4: Figure S2. Loss of BfmRS system activates T6SS-independent killing phenotype of A. baumannii V15 against A. baylyi ADP1 only. Quantitative evaluation of inter-bacterial competition assay displaying a recovered number of prey: (a) E. coli MC4100 and (b) A. baylyi ADP1. Competition was performed with the following strains used as the aggressors: WT, ΔbfmRSΔhcp mutant, ΔbfmRSΔhcp strain complemented with the plasmids pbfmRS or pbfmR. E. coli DH5α was used as a negative non-competitive control to enumerate bacteria numbers if there were no competition. Error bars represent standard deviation. The horizontal lines represent mean value. Values were calculated from at least three independent experiments. ***, p < 0.001; n.s., not significant. [file 12866_2019_1621_MOESM4_ESM.pdf]

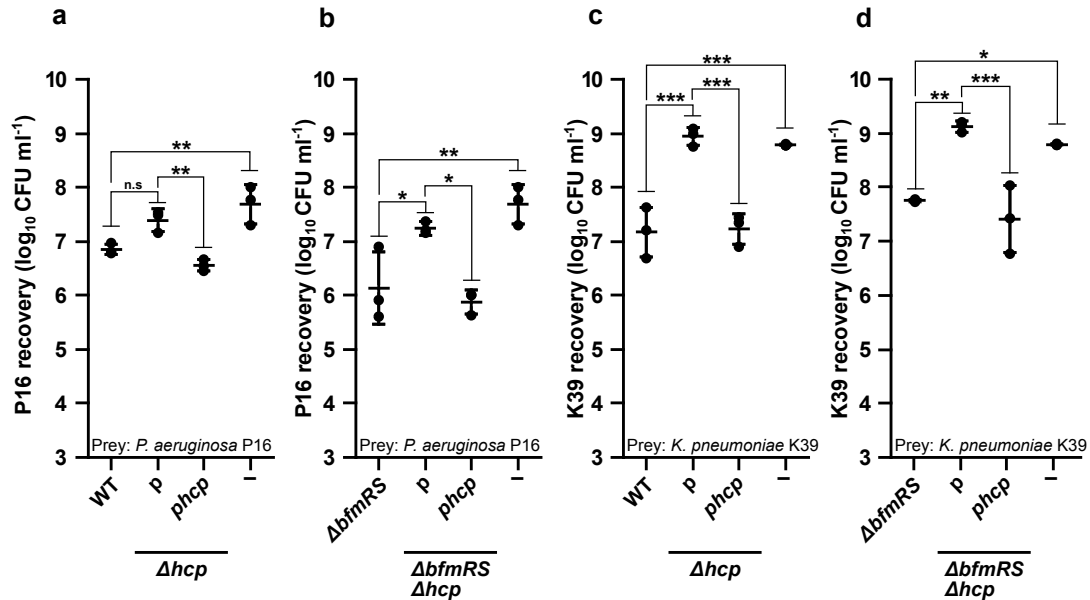

Supplement: Supplementary file 5 — Additional file 5: Figure S3. Quantitative evaluation of inter-bacterial competition assays displaying the recovered numbers of clinical strains that were used as a prey: (a and b) Pseudomonas aeruginosa P16 and (c and d) Klebsiella pneumoniae K39. Competition was performed with the following strains used as the aggressors: WT, ΔbfmRS, Δhcp, ΔbfmRSΔhcp. The hcp mutants were also complemented with the wild-type hcp allele (plasmid phcp). E. coli DH5α was used as a negative non-competitive control to enumerate bacteria numbers if there were no competition. Error bars represent standard deviation. The horizontal lines represent mean value. Values were calculated from at least three independent experiments.*, p < 0.05; **, p < 0.01; ***, p < 0.001; n.s., not significant. The hcp gene in plasmid phcp was induced using IPTG concentration of 0.1 mM. [file 12866_2019_1621_MOESM5_ESM.pdf]

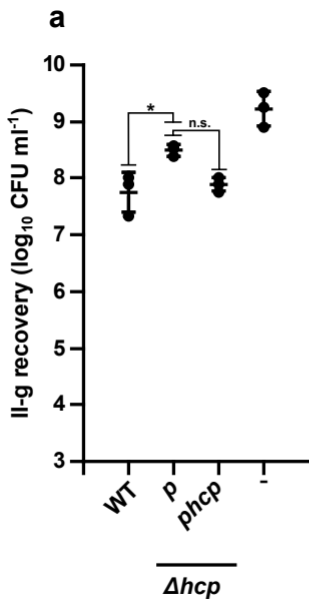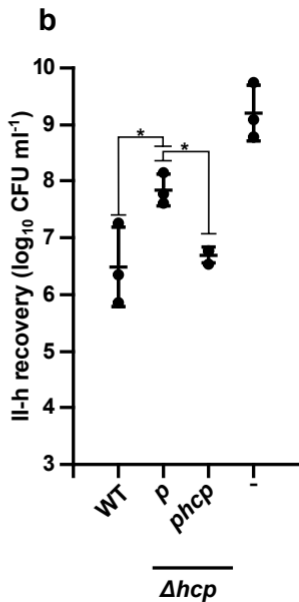

Supplement: Supplementary file 6 — Additional file 6: Figure S4. T6SS is used as the main mechanism for species antagonism by A. baumannii V15 (WT) strain. Quantitative evaluation of inter-bacterial competition assay displaying a recovered number of CDI lacking A. baumannii strains used as a prey: (a) II-g; (b) II-h. Competition was performed with the following strains used as the aggressors: WT, Δhcp mutant, Δhcp mutant complemented with the wild-type hcp allele (plasmid phcp). E. coli DH5α was used as a negative non-competitive control to enumerate bacteria numbers if there were no competition. Error bars represent standard deviation. The horizontal lines represent mean value. Values were calculated from at least three independent experiments.*, p < 0.05; n.s., not significant. Hcp gene in plasmid phcp was induced using IPTG concentration of 0.1 mM. [file 12866_2019_1621_MOESM6_ESM.pdf]

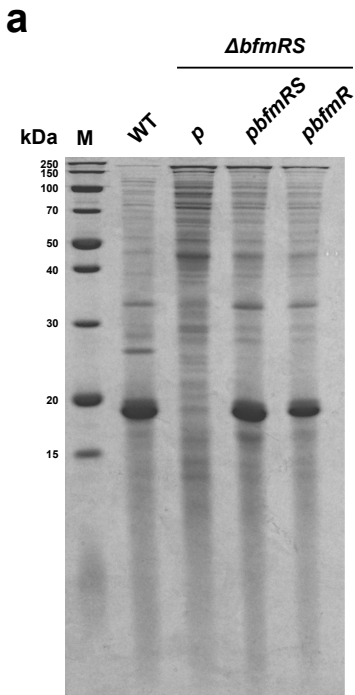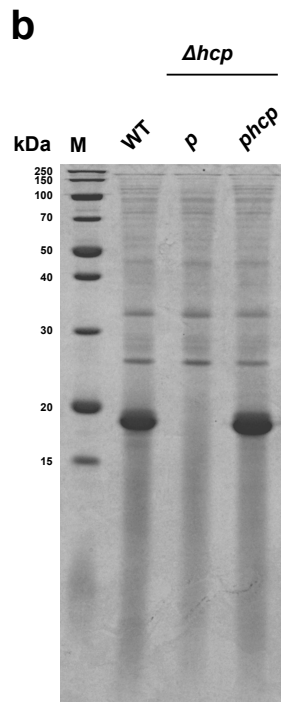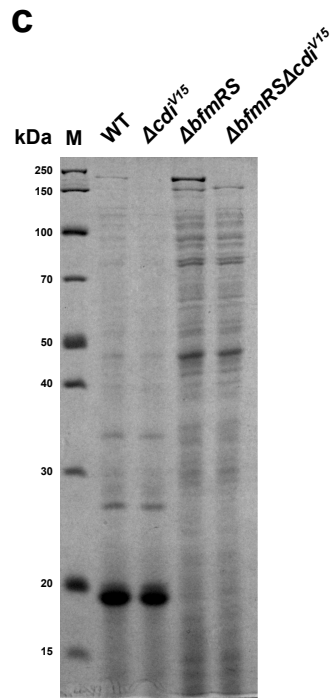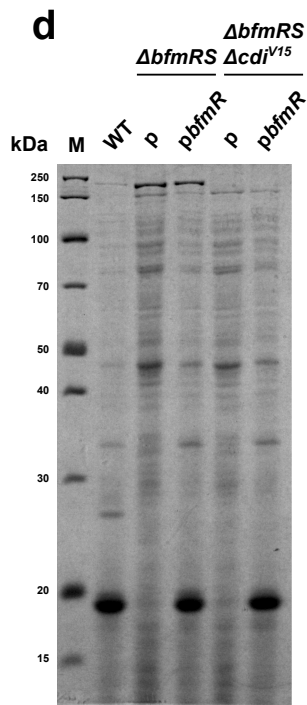

Supplement: Supplementary file 7 — Additional file 7: Figure S5. The whole gels showing TCA-precipitated total protein fraction from culture media. Related to Fig. 1c-d and Fig. 3c-d. Proteins separated by 12.5% (a and b) or 10% (c and d) SDS-PAGE and visualized by staining with Coomassie blue. PageRuler™ Unstained Broad Range Protein Ladder (Thermo Fisher) was used as marker. Numbers on the left of the gels denote molecular mass in kDa. [file 12866_2019_1621_MOESM7_ESM.pdf]
